# LINKING ACES TO LIFE APPRAISAL AMONG OLDER WOMEN IN CORRECTIONAL CUSTODY

**DOI:** 10.1093/geroni/igad104.2169

**Published:** 2023-12-21

**Authors:** Brianna Barnett, Alexander Bishop, Kevin Randall

**Affiliations:** Oklahoma State University, Stillwater, Oklahoma, United States; Oklahoma State University, Stillwater, Oklahoma, United States; Sam Houston State University, Huntsville, Texas, United States

## Abstract

Adverse Childhood Experiences (ACEs), forgiveness, social support, and valuation of life were assessed for older women (45 years of age and older; N = 157) and younger women (those less than 45 years of age: N = 386) in custody in Oklahoma. The Model of Developmental Adaptation led to a path analysis with positive valuation of life or the developmental outcome regressed on proximal influences assessed by mediating variables, social provisions, and forgiveness, and these on the distal predictor, adverse childhood experiences. All endogenous measures were controlled for age, race, marital status, education, and crime type. Using Mplus 8.8 and an estimator that provides parameter estimates and a chi-square test statistic robust to non-normality, the study found that the model fit well for both age group. For the older women, 41% of the developmental outcome, positive valuation of life was explained; whereas for the younger women, 30% was explained. In addition, the standardized path from ACEs to Forgiveness not significant for either the younger or older women. However, for the younger women, ACEs was negatively associated with social provisions (β = -.109, p = .035). In addition, a significant indirect effect was found for the path from ACEs to valuation of life through social provisions (-.03, p = .026, one-tailed). Results have implications relative to how forensic psychologists, correctional case managers, and social workers support the mental health needs of women aging-in-custody. Further discussion will highlight strategies to reduce the impact of ACEs and improve quality-of-life.

